# Serum aspartate aminotransferase, a novel potential biomarker of prognosis in extranodal natural killer/T cell lymphoma, nasal type

**DOI:** 10.3233/CBM-230068

**Published:** 2024-04-15

**Authors:** Ningning Yao, Qing Hou, Yu Liang, Xin Cao, Bochen Sun, Lijuan Wei, Ruifang Sun, Jianzhong Cao

**Affiliations:** a Department of Radiotherapy, Shanxi Province Cancer Hospital, Shanxi Hospital Affiliated to Cancer Hospital, Chinese Academy of Medical Sciences, Cancer Hospital Affiliated to Shanxi Medical University, Taiyuan, Shanxi, China; b Department of Tumor Biobank, Shanxi Province Cancer Hospital, Shanxi Hospital Affiliated to Cancer Hospital, Chinese Academy of Medical Sciences, Cancer Hospital Affiliated to Shanxi Medical University, Taiyuan, Shanxi, China

**Keywords:** Serum aspartate aminotransferase, extranodal natural killer/T cell lymphoma, nasal type, survival, prognosis

## Abstract

**BACKGROUND:**

Aspartate aminotransferase (AST), an indicator of liver cell damage, was related to the prognosis of certain malignant tumors.

**OBJECTIVE:**

This study examined the predictive value of AST in patients with extranodal natural killer/T cell lymphoma (ENKTL).

**METHODS:**

We reviewed 183 cases diagnosed with ENKTL and selected 26 U/L as the optimum cut-off value of AST. We used the univariate and multivariate Cox regression to compare the different AST groups’ overall survival (OS) and progression-free survival (PFS).

**RESULTS:**

Prior to propensity score matching (PSM), Kaplan-Meier analysis showed that patients in the low AST subgroup had better OS and PFS than the high AST subgroup. Multivariate analysis revealed that AST was an independent indicator for prognosis. After PSM, the low AST subgroup maintained a significantly better OS and PFS than the high AST subgroup.

**CONCLUSION:**

AST might represent a significant prognostic marker for ENKTL patients.

## 1. Introduction

Extranodal natural killer T-cell lymphoma, nasal-type (ENKTL), is a rare subtype of non-Hodgkin lymphoma (NHL) with a dismal prognosis [1]. This disease is diversely ethnic and geographic as the incidence is high in Latin America and East Asia [2]. The overall survival of ENKTL has been prolonged by more efficacious treatment strategies; however, their prognosis remains poor [3, 4].

Several predictive models in non-Hodgkin’s lymphoma subtypes remain controversial for ENKTL patients [5, 6]. Some studies optimized the Korean Prognostic Index (KPI) system to present preferable prediction ability by integrating laboratory data [7, 8, 9]. Additionally, some scoring systems have been successively explored for better risk stratifications [10, 11, 12]. Kim et al. proposed the prognostic index (PINK) for ENKTL patients who received L-asparaginase-based regimens. [13]. However, some drawbacks still exist, identification of other validated prognostic markers is essential.

Aspartate aminotransferase (AST), an enzyme with a high level in the liver, was generally tested for liver damage. However, many studies suggest that the AST level is associated with non-liver-related mortality [14, 15, 16, 17]. Tumor cells also produce AST, and the level of AST also correlates with the prognosis of hepatocellular and renal cell carcinoma, breast cancer, and multiple myeloma and high levels of AST indicate poor prognosis [18, 19, 20, 21, 22]. However, few studies on the relationship between AST and lymphoma survival have been illustrated [23]. To our best knowledge, this retrospective study first explored AST’s predictive role in ENKTL patients.

## 2. Methods

### 2.1 Patient collection

In our study, we collected medical records of 183 eligible ENKTL patients from Shanxi Cancer Hospital from January 2002 to December 2018. The inclusion criteria were as follows: (i) confirmed with ENKTL by both pathological diagnosis and immunohistochemistry, (ii) no previous anticancer treatment, (iii) with adequate clinical and follow-up data. Based on the primary tumor site, ENKTL was classified as upper aerodigestive tract NK/T-cell lymphoma (UENKTL) or extra-UENKTL (EUENKTL) [24]. 

### 2.2 Data collection

We collected pretreatment data regarding laboratory examinations, age, sex, ECOG score, serum LDH level, systemic B symptoms, Extranodal invasion sites, regional lymph node involvement, Ann Arbor Staging, and the biochemical profile from the electronic medical record system. We obtained the AST value (U/L) from the hospital laboratory database.

Additionally, we also analyzed IPI (age, performance status, stage, LDH level, and extranodal sites) [25], KPI (stage, LDH level, B symptoms, and regional lymph nodes) [26], PINK (age, Ann Arbor stage, distant lymph-node involvement, and non-nasal type disease) [13] and NRI (age, ECOG Performance Status, Ann Arbor stage, LDH level, and PTI) [27] calculated at diagnosis.

### 2.3 Statistical analysis

The primary endpoints were progression-free survival (PFS) and overall survival time ( OS). We calculated the optimal cut-off value for AST by the change point method (SurvMisc package, R project, version 3.6.1) [28]. According to this value, patients were stratified into high AST and low AST groups. We used the Kaplan-Meier method and log-rank test to show the differences in the survival curves and univariate and multivariate analyses to assess the prognostic factors with hazard ratios recorded with 95% confidence intervals. The regression was verified using the 10-fold cross-validation with a seed number of 2022. We considered P< 0.05 to be statistically significant in all analyses. Propensity score matching (PSM) was performed using the *Matching* package to minimize selection bias, using nearest neighbor matching (1:1) with a caliper distance of 0.02. Time-dependent ROC was performed using the *timeROC* to calculate the area under the curve (AUC). We conducted the statistical analyses using IBM SPSS 20.0, GraphPad Prism 8, and R software 3.6.1. 

**Table 1:** Clinicopathological features of 183 patients according to the AST

Characteristics	Number of patients (%)	AST < 26 U/L	AST ⩾ 26 U/L	P value
Age				0.318
⩽ 60 y	147(80.33%)	87(82.86%)	60(76.92%)	
> 60 y	36(19.67%)	18(17.14%)	18(23.08%)	
Sex				0.040
Male	144(78.69%)	77(73.33%)	67(85.90%)	
Female	39(21.31%)	28(26.67%)	11(14.10%)	
ECOG score				0.169
0–1	147(80.33%)	88(83.81%)	59(75.64%)	
⩾ 2	36(19.67%)	17(16.19%)	19(24.36%)	
Ann Arbor Stage				0.271
I–II	141(77.05%)	84(80.00%)	57(73.08%)	
III–IV	42(22.95%)	21(20.00%)	21(26.92%)	
B symptoms				0.032
No	117(63.93%)	74(70.48%)	43(55.13%)	
Yes	66(36.07%)	31(29.52%)	35(44.87%)	
Extranodal sites of involvement				0.146
< 2	158(86.34%)	94(89.52%)	64(82.05%)	
⩾ 2	25(13.66%)	11(10.48%)	14(17.95%)	
Regional lymph node involvement				0.002
Yes	66(36.07%)	28(26.67%)	38(48.72%)	
No	117(63.93%)	77(73.33%)	40(51.28%)	
Subtype				0.269
UNKTL	169(92.35%)	95(90.48%)	74(94.87%)	
EUNKTL	14(7.65%)	10(9.52%)	4(5.13%)	
Serum LDH				< 0.001
⩽ 245 u/l	129(70.49%)	96(91.43%)	33(42.31%)	
> 245 u/l	54(29.51%)	9(8.57%)	45(57.69%)	
Hemoglobin				0.566
⩽ 120 g/l	43(23.50%)	23(21.90%)	20(25.64%)	
> 120 g/l	140(76.50%)	82(78.10%)	58(74.36%)	
ALT				< 0.001
⩽ 25	100(54.64%)	84(80.00%)	16(20.51%)	
> 25	83(45.36%)	21(20.00%)	62(79.49%)	
AST/ALT				< 0.001
Low	111(60.66%)	83(79.05%)	28(35.90%)	
High	72(39.34%)	22(20.95%)	50(64.10%)	
IPI				0.001
0–1	132(72.13%)	86(81.90%)	46(58.97%)	
2–5	51(27.87%)	19(18.10%)	32(41.03%)	
KPI				< 0.001
0–1	116(63.39%)	83(79.05%)	33(42.31%)	
2–4	67(36.61%)	22(20.95%)	45(57.69%)	
PINK				0.897
0–1	147(80.33%)	84(80.00%)	63(80.77%)	
2–4	36(19.67%)	21(20.00%)	15(19.23%)	
NRI				< 0.001
0–1	86(46.99%)	63(60.00%)	23(29.49%)	
2–6	97(53.01%)	42(40.00%)	55(70.51%)	
RT				0.526
No	61(33.33%)	33(31.43%)	28(35.90%)	
Yes	122(66.67%)	72(68.57%)	50(64.10%)	
L-Asp				0.189
No	100(54.64%)	53(50.48%)	47(60.26%)	
Yes	83(45.36%)	52(49.52%)	31(39.74%)	
Liver disease				0.316
No	174(95.08%)	102(93.60%)	72(97.30%)	
Yes	9(4.92%)	7(6.40%)	2(2.70%)	
SCT				1.000
No	179(97.81%)	107(98.20%)	72(97.30%)	
Yes	4(2.19%)	2(1.80%)	2(2.70%)	

Abbreviation: LDH, lactate dehydrogenase; IPI, International Prognostic Index; KPI, Korean Prognostic Index; PINK, Prognostic index of natural killer lymphoma; NRI, nomogram-revised risk index; RT, radiotherapy; L-Asp, L-Asparaginase; SCT, allogeneic hematopoietic stem cell transplantation.

### 2.4 Ethical approval

The study was approved by the ethics committees at Shanxi Province Cancer Hospital, Shanxi Hospital Affiliated to Cancer Hospital, Chinese Academy of Medical Sciences, Cancer Hospital Affiliated to Shanxi Medical University and the review board approved to waive the requirement for informed consent. (Number: 2019091). Data collection was carried out via electronic medical records and entered in an anonymized databank. All analyses were performed in accordance with the relevant guidelines and regulations.

**Figure 1.**
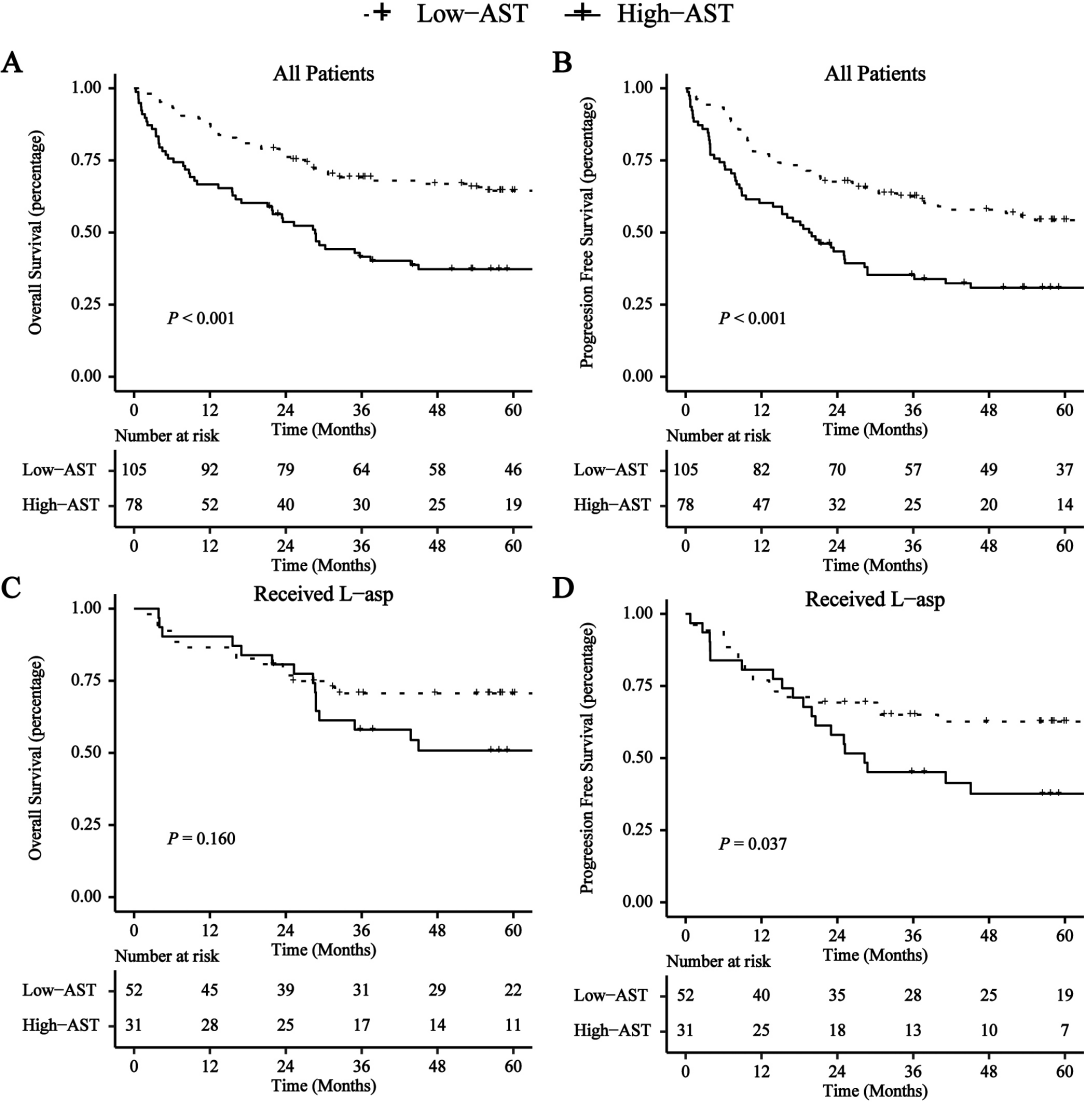
Survival curves for OS and PFS based on AST (< 26, ⩾ 26) in patients with ENKTL. A for OS and B for PFS in all patients, C for OS and D for PFS in patients received L-asp.

## 3. Results

### 3.1 Patients’ characteristics

Table 1 shows the clinical parameters of the patients. We obtained AST before treatment. At the time of diagnosis, the median AST was 24 U/L, and the most discriminative cut-off value was 26 U/L. We compared baseline clinical characteristics of high AST patients (AST⩾ 26 U/L) with those of low AST patients (AST < 26 U/L). We found no significant differences in age, sex, ECOG score, Ann Arbor Stage, extranodal sites of involvement, subtype, hemoglobin level, ALT level, radiotherapy, and L-Asp regimen. Patients with high AST levels tended to have more frequent B symptoms, regional lymph node involvement, and elevated serum LDH level.

**Table 2:** Univariate and multivariate analysis of prognostic factors for PFS and OS in patients with ENKTL

	OS				PFS			
	Univariate analysis		Multivariate analysis		Univariate analysis		Multivariate analysis	
	P value		HR (95%CI)	P value	P value		HR (95%CI)	P value
Age	0.	041^*^			0.	296		
Sex	0.	330			0.	192		
ECOG score	< 0.	001^*^	2.128 (1.309–3.457)	0.002	< 0.	001^*^	1.862 (1.163–2.980)	0.010
Ann Arbor Stage	0.	101			0.	571		
B symptoms	0.	735			0.	820		
Extranodal sites of involvement	0.	112			0.	431		
Regional lymph node involvement	0.	035^*^			0.	080^*^		
Subtype	0.	932			0.	782		
KPI	0.	006^*^			0.	057		
IPI	< 0.	001^*^			0.	036^*^		
PINK	0.	065			0.	284		
NRI	< 0.	001^*^	2.002 (1.230–3.260)	0.005	< 0.	001^*^	1.618 (1.051–2.490)	0.029
Serum LDH	0.	001^*^			0.	020^*^		
Hb	0.	135			0.	131		
AST	< 0.	001^*^	1.999 (1.290–3.100)	0.002	< 0.	001^*^	1.823 (1.223–2.716)	0.003
ALT	0.	066			0.	093		
AST/ALT	0.	016			0.	103		
RT	0.	001^*^	0.581 (0.371–0.909)	0.017	< 0.	001^*^	0.592 (0.387–0.905)	0.015
L-Asp	0.	005^*^	0.552 (0.352–0.864)	0.009	0.	022^*^	0.655 (0.438–0.978)	0.039
								

### 3.2 Survival analysis

For all the enrolled patients, the median survival time was 137 months. Forty-seven percent of patients (n= 86) had died by the time of follow-up. At 3 and 5 years were 57.3% and 52.8%, and PFS at 3 and 5 years were 51.0% and 44.3%, respectively. Patients with low AST had better 5-year OS (64.5% vs. 37.3%; P< 0.001) and PFS (54.3% vs. 35.3%; P< 0.001) than patients with high AST (P< 0.001; Fig. 1). Table 2 shows the univariate and multivariate analysis results for OS and PFS. Univariate analysis identified that the OS of patients with high AST, elder age, ECOG score ⩾2, regional lymph node involvement, KPI score ⩾ 2, IPI score ⩾ 2, NRI score ⩾ 2, elevated serum LDH level, ALT/AST, radiotherapy, and L-Asp-based chemotherapy untreated was significantly shorter (P< 0.05). In the forward LR Cox regression model, all the statistically significant factors in the univariate analysis were involved. Independent prognostic factors of OS were AST (HR = 1.999, 95%CI = 1.290–3.100, P= 0.002), ECOG score (HR = 2.128, 95%CI = 1.309–3.457, P= 0.002), NRI score (HR = 2.002, 95%CI = 1.230–3.260, P= 0.005), radiotherapy (HR = 0.581, 95%CI = 0.371–0.909, P= 0.017), and L-Asp-based chemotherapy (HR = 0.552, 95%CI = 0.352–0.864, P= 0.009). Similarly, AST was independently predictive of PFS (HR = 1.823, 95%CI = 1.223–2.716, P= 0.003).

In the cohort of patients, 45.4% (n= 83) patients received L-Asp-based chemotherapy. For this group, the level of AST was significant against PFS (P= 0.037) but not against OS (P= 0.160). The 3-year and 5-year PFS of low AST group (n= 52) are 65.0% and 62.6%, and those of the high AST group (n= 31) are 45.2% and 37.6% (Fig. 1), respectively. The difference was not significant in OS (70.6%, 70.6% vs. 58.1%, 50.8%) (Fig. 1).

### 3.3 Propensity score matching analysis

**Figure 2.**
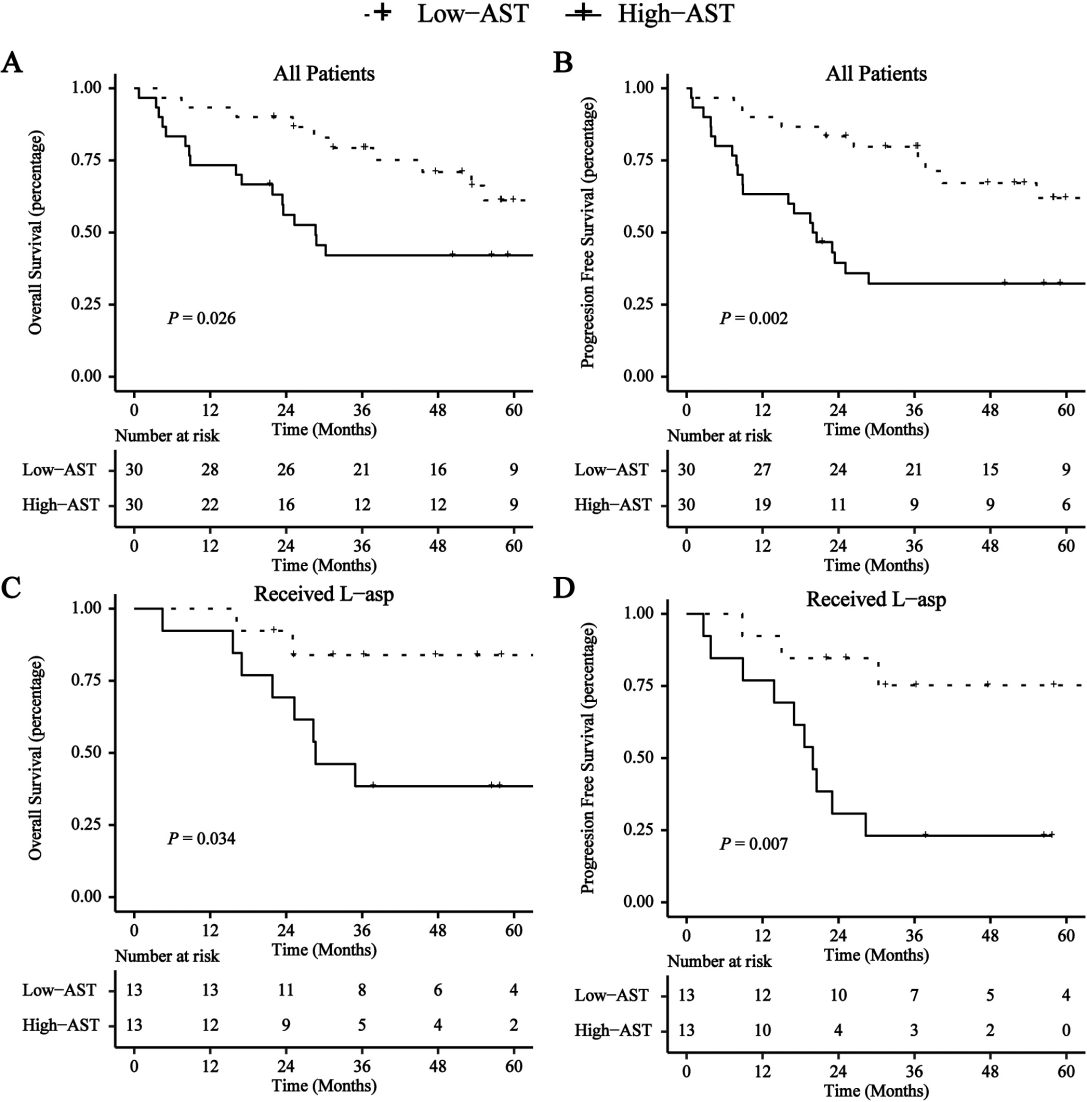
Survival curves for OS and PFS based on AST (< 26, ⩾ 26) after PSM. A for OS and B for PFS in all patients , C for OS and D for PFS in patients received L-asp.

To decrease the effect of confounding factors, we performed PSM analysis using the factors including LN, LDH, ALTAST, ALT, IPI, KPI, NRI, L-Asp. After PSM, the differences for all covariates between low- (n= 30) and high (n= 30) AST group were eliminated (Table 3). In the matched group, the 3-year and 5-year OS and PFS are 60.7%, 52.3%, 56.1%, 47.7%, respectively. Similar to the entire patients, there was a significant difference for OS (P= 0.026) and PFS (P= 0.002) between low AST and high AST (Fig. 2). The 3-year and 5-year OS of the low AST group (79.3%, 61.1%) are longer than those of the high AST group (42.1%, 42.1%). The 3-year and 5-year PFS of the low AST group (79.7%, 62.0%) are longer than those of the high AST group (32.3%, 32.3%).

**Table 3:** Clinicopathological features of 60 patients according to the AST after PSM

Characteristics	Number of patients (%)	AST < 26 U/L	AST ⩾ 26U/L	P Value
Age				0.739
⩽ 60 y	49(81.67%)	24(80.00%)	25(83.33%)	
> 60 y	11(18.33%)	6(20.00%)	5(16.67%)	
Sex				1.000
Male	53(88.33%)	26(86.67%)	27(90.00%)	
Female	7(11.67%)	4(13.33%)	3(10.00%)	
ECOG score				0.095
0–1	49(81.67%)	27(90.00%)	22(73.33%)	
⩾ 2	11(18.33%)	3(10.00%)	8(26.67%)	
Ann Arbor Stage				0.766
I–II	45(75.00%)	22(73.33%)	23(76.67%)	
III–IV	15(25.00%)	8(26.67%)	7(23.33%)	
B symptoms				0.426
No	37(61.67%)	20(66.67%)	17(56.67%)	
Yes	23(38.33%)	10(33.33%)	13(43.33%)	
Extranodal sites of involvement				1.000
< 2	52(86.67%)	26(86.67%)	26(86.67%)	
⩾ 2	8(13.33%)	4(13.33%)	4(13.33%)	
Regional lymph node involvement				0.184
Yes	23(38.33%)	9(30.00%)	14(46.67%)	
No	37(61.67%)	21(70.00%)	16(53.33%)	
Subtype				1.000
UNKTL	59(98.33%)	29(96.67%)	30(100.00%)	
EUNKTL	1(1.67%)	1(3.33%)	0(0.00%)	
Serum LDH				0.108
⩽ 245 u/l	38(63.33%)	22(73.33%)	16(53.33%)	
> 245 u/l	22(36.67%)	8(26.67%)	14(46.67%)	
Hemoglobin				0.317
⩽ 120 g/l	11(18.33%)	4(13.33%)	7(23.33%)	
> 120 g/l	49(81.67%)	26(86.67%)	23(76.67%)	
ALT				0.787
⩽ 25	21(35.00%)	10(33.33%)	11(36.67%)	
> 25	39(65.00%)	20(66.67%)	19(63.33%)	
AST/ALT				0.436
Low	27(45.00%)	15(50.00%)	12(40.00%)	
High	33(55.00%)	15(50.00%)	18(60.00%)	
IPI				0.774
0–1	43(71.67%)	21(70.00%)	22(73.33%)	
2–5	17(28.33%)	9(30.00%)	8(26.67%)	
KPI				0.194
0–1	33(55.00%)	19(63.33%)	14(46.67%)	
2–4	27(45.00%)	11(36.67%)	16(53.33%)	
PINK				0.317
0–1	49(81.67%)	23(76.67%)	26(86.67%)	
2–4	11(18.33%)	7(23.33%)	4(13.33%)	
NRI				0.118
0–1	26(43.33%)	16(53.33%)	10(33.33%)	
2–6	34(56.67%)	14(46.67%)	20(66.67%)	
RT				1.000
No	18(30.00%)	9(30.00%)	9(30.00%)	
Yes	42(70.00%)	21(70.00%)	21(70.00%)	
L-Asp				1.000
No	32(53.33%)	16(53.33%)	16(53.33%)	
Yes	28(46.67%)	14(46.67%)	14(46.67%)	

Abbreviation: LDH, lactate dehydrogenase; IPI, International Prognostic Index; KPI, Korean Prognostic Index; PINK, Prognostic index of natural killer lymphoma; NRI, nomogram-revised risk index; RT, radiotherapy; L-Asp, L-Asparaginase.

We performed PSM analysis using factors including LDH, ALT/AST, ALT, KPI in the L-Asp-based chemotherapy group. After PSM, the differences for all covariates between the low- (n= 13) and high (n= 13) AST group were eliminated (Supplemental Table 1). In the matched group, the 3-year and 5-year OS and PFS are 59.4%, 59.4%, 48.6%, 48.6%, respectively. The OS (P= 0.034) and PFS (P= 0.007) were significant differences between low AST and high AST in the matched group. The 3-year and 5-year OS of the low AST group (83.9%, 83.9%) are longer than those of the high AST group (38.5%, 38.5%). The 3-year and 5-year PFS of the low AST group (75.2%, 75.2%) are longer than those of the high AST group (23.1%, 23.1%) (Fig. 2).

### 3.4 Subgroup analyses

**Figure 3.**
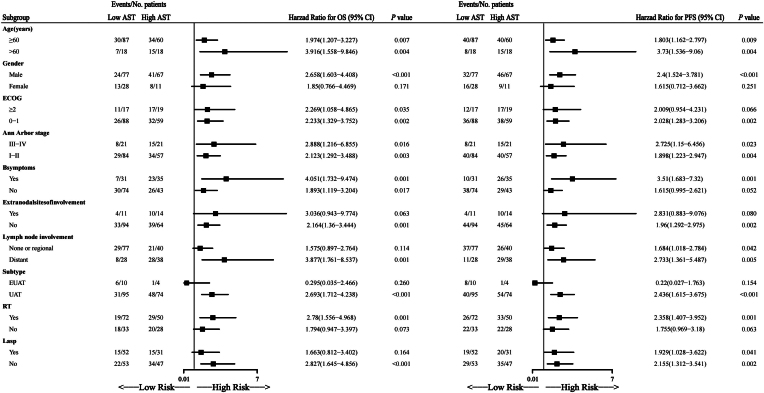
Forest plot depicting the HRs of AST in different risk subgroups for OS and PFS.

We performed a subgroup analysis based on patients’ baseline characteristics and treatment, including age, gender, ECOG score, Ann Arbor stage, B symptoms, extranodal involvement site, regional lymph node involvement, subtype, RT, and L-Asp. Figure 3 showed that OS and PFS were shorter in almost all subgroups with a high AST level than those with a low AST. The HRs were 1.575 to 3.916 and 1.803 to 3.730, respectively.

**Figure 4.**
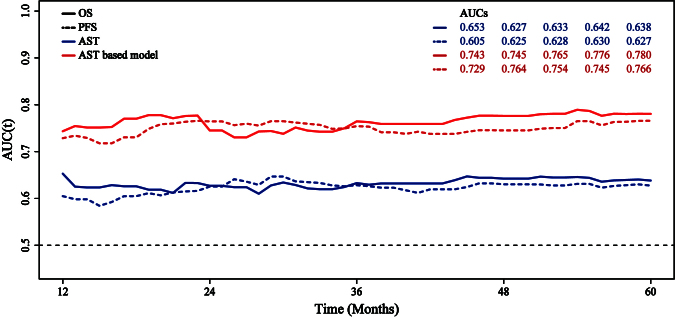
Time-dependent ROC curve for predicting ENKTL patients’ OS.

### 3.5 Prognostic value of AST

Accordingly, time-dependent receiver operating characteristics (ROC) curves revealed that AST was a powerful predictor for ENKTL with the area under the curve (AUC) was 0.653, 0.627, 0.633, 0.642, and 0.638 for OS at 12, 24, 36, 48, and 60 months, and 0.605, 0.625, 0.628, 0.630, and 0.627 for PFS at 12, 24, 36, 48, and 60 months, respectively (Fig. 4).

The time-dependent AUCs calculated with 5-fold cross validation was used to assess the performance of the AST on the training cohort and the validation cohort, respectively. When predicting 12, 24, 36, 48, and 60 months OS, the average AUC in the training cohort was 0.654, 0.627, 0.633, 0.643, and 0.639, respectively, and 0.675, 0.627, 0.637, 0.649, and 0.646 in the validation cohort, respectively. When predicting PFS at 12, 24, 36, 48 and 60 months, the average AUC was 0.605, 0.626, 0.628, 0.631, and 0.628 in the training set, and 0.605, 0.628, 0.631, 0.638, and 0.631 in the validation set, respectively (Supplemental Table 2).

AST-based model for predicting OS (Fig. S1) and PFS (Fig. S2) was built based on the multivariate Cox regression model. The time-dependent ROC curve showed that the AST-based model could more effectively predict the OS and PFS of patients. The time-dependent ROC curves revealed that AST was a powerful predictor for ENKTL with the area under the curve (AUC) was 0.743, 0.745, 0.765, 0.776, and 0.780 for OS at 12, 24, 36, 48, and 60 months, and 0.729, 0.764, 0.754, 0.745, and 0.766 for PFS at 12, 24, 36, 48, and 60 months, respectively (Fig 4).

## 4. Discussion

In this study, we first presented that the serum AST was a prognostic indicator for patients with ENKTL. Recently, some studies reported that AST was increasingly associated with the outcomes of some malignancies, such as non-small cell lung cancer, multiple myeloma, breast cancer, and pancreatic cancer [19, 22, 30, 31]. Furthermore, AST could be a prognostic indicator integrated with ALT, another important circulating transaminase [32, 33]. The ratio of AST to lymphocyte also plays an essential part in tumor prognosis [18, 34]. A study in non-Hodgkin lymphoma showed that higher AST levels predicted a worse prognosis in DLBCL [23]. ENKTL is also a subtype of non-Hodgkin lymphoma, and we hypothesized that AST may be associated with the prognosis of NKT patients. Our results in ENKTL patients were consistent with this finding.

The results of a large-scale study including 416,122 patients showed that elevated AST was not only significantly associated with death from all causes of non-liver disease (⩾ 40 vs. 15–24, HR: 1.36, 95% CI: 1.27–1.46), but associated with poor prognosis in patients with non-liver cancer (⩾ 40 vs. 15–24, HR: 1.45, 95% CI: 1.29–1.62). However, the underlying mechanism of the relationship between AST and the survival of ENKTL patients needs to be more precise. AST is an essential enzyme in gluconeogenesis and amino acid metabolites. Since most cancer cells produce ATP through glycolysis which is necessary for maintaining survival, growth, and invasion, there is a strong link between AST and carcinogenesis [35].

Many studies conventionally evaluated the predictive values of IPI, KPI, and Ann Arbor scores for ENKTL patients. However, the application values still need to be further discussed. Previous studies have shown that most patients were categorized as the low-risk group based on IPI and KPI score and classified as early-stage based on the Ann Arbor Staging System [36]. The disproportionate distribution failed to achieve precise prediction and appropriate clinical guidance. AST helped identify patients with unfavorable outcomes in the low-risk group categorized by the above score systems.

In addition, our finding indicated that higher AST was associated with unfavorable OS and PFS in patients who received L-Asp-based chemotherapy. The efficacy of conventional CHOP (cyclophosphamide, doxorubicin, vincristine, and prednisone) or CHOP-based chemotherapy was limited because of the resistance even when followed by radiotherapy [37, 38]. A meta-analysis showed that L-Asp-based chemotherapy significantly improved complete response (CR) and overall response rate (ORR) of early-stage and advanced-stage ENKTL patients compared with L-Asp-absent regimen [39] and was reported to have more than 80% response rates in patients with refractory or relapsed ENKTL [40, 41]. In this study, we also showed that AST was an independent prognostic factor in receiving L-Asp-based chemotherapy patients.

This study has the following limitations. Due to the .retrospective analysis of a limited number of patients, we need to determine the diagnostic value and further validation through large-scale prospective studies. Besides, further investigations are required to delineate the mechanisms.

## 5. Conclusion

AST could be an influential prognostic factor for patients with ENKTL.

## Funding

This research was funded by the Applied Basic Research Projects of Shanxi Province [No. 20210302124 598], and the Fund Program for the Scientific Activities of Selected Returned Overseas Professionals in Shanxi Province (Department of Resource and Social Security of Shanxi Province No. [2019]1176), the Research Project Supported by Shanxi Scholarship Council of China No. [2022]210, the Key Research and Development (R&D) Projects of Shanxi Province [No. 201803D421054], Wu Jieping Medical Foundation No. 320.6750.2022-1-53, Lianyungang Yixing Medical Health Foundation, and the Four “Batches” Innovation Project of Invigorating Medical through Science and Technology of Shanxi Province No. [2022]37. 

## Author contributions

Conception: Cao J, Sun R.

Interpretation or analysis of data: Yao NN, Hou Q, Liang Y, Cao X, Sun B, Wei L.

Preparation of the manuscript: Yao NN, Hou Q, Liang Yu.

Revision for important intellectual content: Sun R, Cao J.

## Data availability statement

The data of this study are available from the corresponding author upon reasonable request.

## Supplementary data

The supplementary files are available to download from http://dx.doi.org/10.3233/CBM-230068. 

## Supplementary Material

Supplementary table and Figure
