# Supplementary material for: Serum aspartate aminotransferase, a novel potential biomarker of prognosis in extranodal natural killer/T cell lymphoma, nasal type
Source: Cancer Biomark. 2024 Apr 15;39(4):265–75. doi: 10.3233/CBM-230068 (PMC11191476; doi:10.3233/CBM-230068)

**Supplemental Table 1** Clinicopathological features of patients who received L-asp before and after PSM

| Characteristics | Total Number of patients (%) | Before PSM | | | After PSM | | |
| --- | --- | --- | --- | --- | --- | --- | --- |
|  |  | AST<26U/L | AST≥26U/L | *P* Value | AST<26U/L | AST≥26U/L | *P* Value |
| All patients |  |  |  |  |  |  |  |
| Age |  |  |  | 0.758 |  |  | 1.000 |
| ≤60 y | 70(84.3%) | 43(82.69%) | 27(87.1%) |  | 11(84.62%) | 11(84.62%) |  |
| >60 y | 13(15.7%) | 9(17.31%) | 4(12.9%) |  | 2(15.38%) | 2(15.38%) |  |
| Sex |  |  |  | 0.187 |  |  | 1.000 |
| Male | 66(79.5%) | 39(75%) | 27(87.1%) |  | 13.00(100%) | 12(92.31%) |  |
| Female | 17(20.5%) | 13(25%) | 4(12.9%) |  | 0.00(0%) | 1(7.69%) |  |
| ECOG score |  |  |  | 1.000 |  |  | 0.593 |
| 0-1 | 72(86.7%) | 45(86.54%) | 27(87.1%) |  | 12(92.31%) | 10(76.92%) |  |
| ≥2 | 11(13.3%) | 7(13.46%) | 4(12.9%) |  | 1(7.69%) | 3(23.08%) |  |
| Ann Arbor Stage |  |  |  | 0.779 |  |  | 0.673 |
| I-II | 63(75.9%) | 40(76.92%) | 23(74.19%) |  | 10(76.92%) | 8(61.54%) |  |
| III-IV | 20(24.1%) | 12(23.08%) | 8(25.81%) |  | 3(23.08%) | 5(38.46%) |  |
| B symptoms |  |  |  | 0.396 |  |  | 0.431 |
| No | 53(63.9%) | 35(67.31%) | 18(58.06%) |  | 7(53.85%) | 5(38.46%) |  |
| Yes | 30(36.1%) | 17(32.69%) | 13(41.94%) |  | 6(46.15%) | 8(61.54%) |  |
| Extranodal sites of involvement |  |  |  | 0.350 |  |  | 0.645 |
| <2 | 71(85.5%) | 46(88.46%) | 25(80.65%) |  | 11(84.62%) | 9(69.23%) |  |
| ≥2 | 12(14.5%) | 6(11.54%) | 6(19.35%) |  | 2(15.38%) | 4(30.77%) |  |
| Regional lymph node involvement |  |  |  | 0.059 |  |  | 0.234 |
| No | 51(61.4%) | 36(69.23%) | 15(48.39%) |  | 9(69.23%) | 6(46.15%) |  |
| Yes | 32(38.6%) | 16(30.77%) | 16(51.61%) |  | 4(30.77%) | 7(53.85%) |  |
| Subtype |  |  |  | 0.722 |  |  | 1.000 |
| UNKTL | 74(89.2%) | 47(90.38%) | 27(87.1%) |  | 13(100.00%) | 12(92.31%) |  |
| EUNKTL | 9(10.8%) | 5(9.62%) | 4(12.9%) |  | 0(0.00%) | 1(7.69%) |  |
| Serum LDH |  |  |  | <0.001 |  |  | 0.234 |
| ≤245 u/l | 63(75.9%) | 48(92.31%) | 15(48.39%) |  | 9(69.23%) | 6(46.15%) |  |
| >245 u/l | 20(24.1%) | 4(7.69%) | 16(51.61%) |  | 4(30.77%) | 7(53.85%) |  |
| Hemoglobin |  |  |  | 0.911 |  |  | 0.593 |
| ≤120 g/l | 22(26.5%) | 14(26.92%) | 8(25.81%) |  | 1(7.69%) | 3(23.08%) |  |
| >120 g/l | 61(73.5%) | 38(73.08%) | 23(74.19%) |  | 12(92.31%) | 10(76.92%) |  |
| ALT |  |  |  | <0.001 |  |  | 1.000 |
| ≤25 | 37(44.6%) | 11(21.15%) | 26(83.87%) |  | 11(84.62%) | 11(84.62%) |  |
| >25 | 46(55.4%) | 41(78.85%) | 5(16.13%) |  | 2(15.38%) | 2(15.38%) |  |
| AST/ALT |  |  |  | <0.001 |  |  | 0.691 |
| Low | 47(56.6%) | 38(73.08%) | 9(29.03%) |  | 5(38.46%) | 6(46.15%) |  |
| High | 36(43.4%) | 14(26.92%) | 22(70.97%) |  | 8(61.54%) | 7(53.85%) |  |
| IPI |  |  |  | 0.061 |  |  | 0.420 |
| 0-1 | 63(75.9%) | 43(82.69%) | 20(64.52%) |  | 9(69.23%) | 7(53.85%) |  |
| 2-5 |  | 20(24.1%) | 9(17.31%) |  | 4(30.77%) | 6(46.15%) |  |
| KPI |  |  |  | 0.036 |  |  | 0.431 |
| 0-1 | 57(68.7%) | 40(76.92%) | 17(54.84%) |  | 7(53.85%) | 5(38.46%) |  |
| 2-4 | 26(31.3%) | 12(23.08%) | 14(45.16%) |  | 6(46.15%) | 8(61.54%) |  |
| PINK |  |  |  | 0.256 |  |  | 1.000 |
| 0-1 | 67(80.7%) | 40(76.92%) | 27(87.1%) |  | 10(76.92%) | 10(76.92%) |  |
| 2-4 | 16(19.3%) | 12(23.08%) | 4(12.9%) |  | 3(23.08%) | 3(23.08%) |  |
| NRI |  |  |  | 0.444 |  |  | 0.42 |
| 0-1 | 42(50.6%) | 28(53.85%) | 14(45.16%) |  | 6(46.15%) | 4(30.77%) |  |
| 2-6 | 41(49.4%) | 24(46.15%) | 17(54.84%) |  | 7(53.85%) | 9(69.23%) |  |
| RT |  |  |  | 0.923 |  |  | 0.202 |
| Yes | 53(63.9%) | 33(63.46%) | 20(64.52%) |  | 11(84.62%) | 7(53.85%) |  |
| No | 30(36.1%) | 19(36.54%) | 11(35.48%) |  | 2(15.38%) | 6(46.15%) |  |

**Abbreviation: *LDH***, lactate dehydrogenase; ***IPI***, International Prognostic Index; ***KPI***, Korean Prognostic Index; ***PINK***, Prognostic index of natural killer lymphoma; ***NRI***, nomogram-revised risk index; ***RT***, radiotherapy; ***L-Asp***, L-Asparaginase; ***SCT***, allogeneic hematopoietic stem cell transplantation.

**Supplemental Table 2** The time-dependent-AUCs of the AST in 5-fold cross-validation

|  | **Months** | | **Training** | | | | | | | | | | | | **Validation** | | | | | | |
| --- | --- | --- | --- | --- | --- | --- | --- | --- | --- | --- | --- | --- | --- | --- | --- | --- | --- | --- | --- | --- | --- |
|  |  |  | **Mean** | | **Min** | | **1st Qu** | | **Median** | | **3rd Qu** | | **Max** | | **Mean** | | **Min** | **1st Qu** | **Median** | **3rd Qu** | **Max** |
| OS | 12 | | 0.654 | | 0.640 | | 0.646 | | 0.649 | | 0.661 | | 0.673 | | 0.675 | | 0.581 | 0.657 | 0.657 | 0.685 | 0.797 |
|  | 24 | | 0.627 | | 0.589 | | 0.631 | | 0.637 | | 0.639 | | 0.640 | | 0.627 | | 0.573 | 0.585 | 0.609 | 0.612 | 0.755 |
|  | 36 | | 0.633 | | 0.601 | | 0.629 | | 0.643 | | 0.646 | | 0.647 | | 0.637 | | 0.569 | 0.579 | 0.641 | 0.649 | 0.746 |
|  | 48 | | 0.643 | | 0.620 | | 0.644 | | 0.644 | | 0.652 | | 0.654 | | 0.649 | | 0.598 | 0.635 | 0.639 | 0.657 | 0.718 |
|  | 60 | | 0.639 | | 0.625 | | 0.633 | | 0.639 | | 0.648 | | 0.649 | | 0.646 | | 0.601 | 0.639 | 0.645 | 0.662 | 0.682 |
| PFS | 12 | | 0.605 | | 0.595 | | 0.599 | | 0.608 | | 0.611 | | 0.612 | | 0.605 | | 0.562 | 0.581 | 0.606 | 0.634 | 0.641 |
|  | 24 | | 0.626 | | 0.618 | | 0.619 | | 0.624 | | 0.625 | | 0.642 | | 0.628 | | 0.542 | 0.627 | 0.653 | 0.656 | 0.661 |
|  | 36 | | 0.628 | | 0.618 | | 0.621 | | 0.624 | | 0.635 | | 0.644 | | 0.631 | | 0.561 | 0.616 | 0.647 | 0.657 | 0.675 |
|  | 48 | | 0.631 | | 0.616 | | 0.620 | | 0.629 | | 0.640 | | 0.648 | | 0.638 | | 0.584 | 0.593 | 0.658 | 0.667 | 0.687 |
|  | 60 | | 0.628 | | 0.614 | | 0.623 | | 0.631 | | 0.632 | | 0.638 | | 0.631 | | 0.593 | 0.622 | 0.623 | 0.641 | 0.677 |


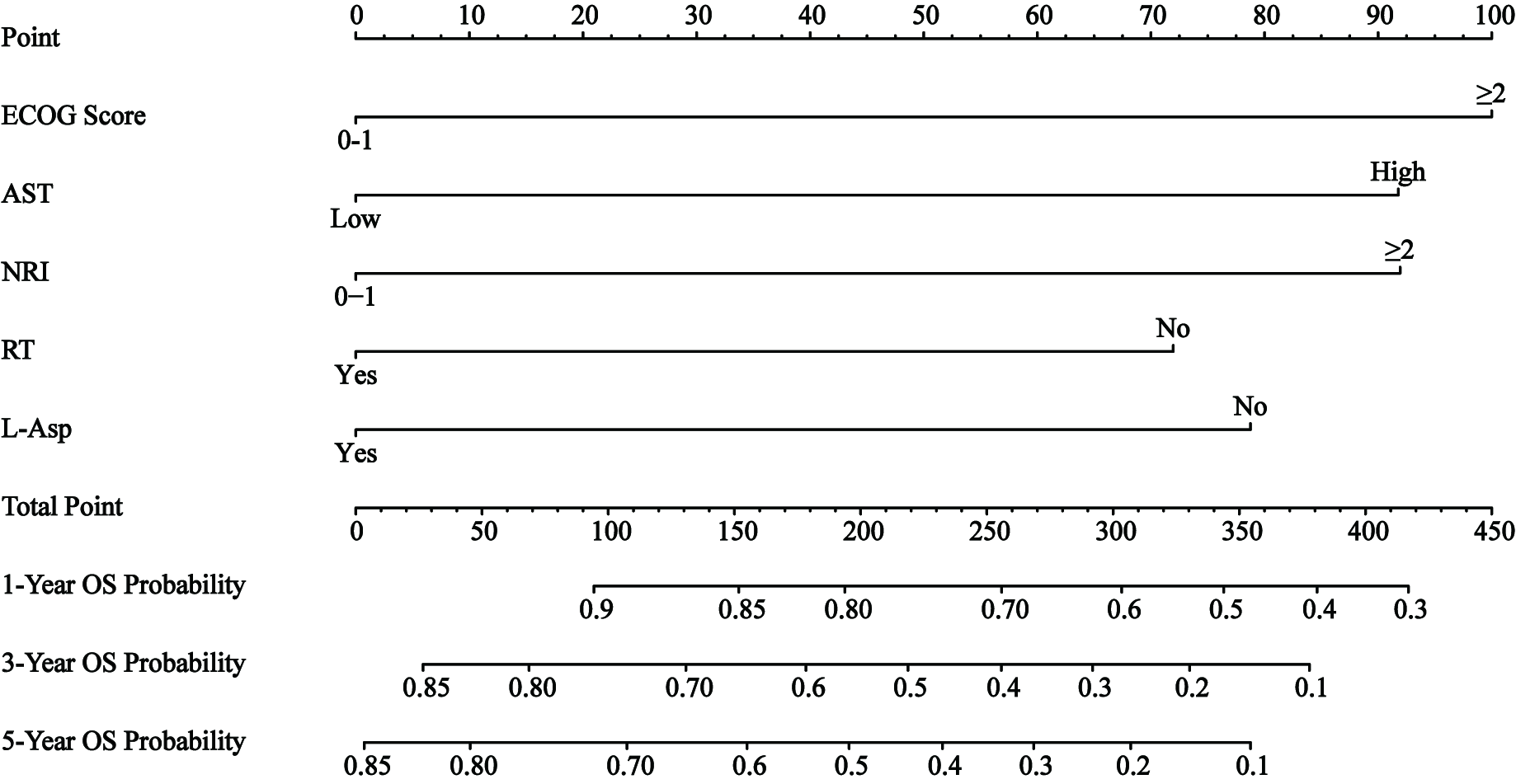

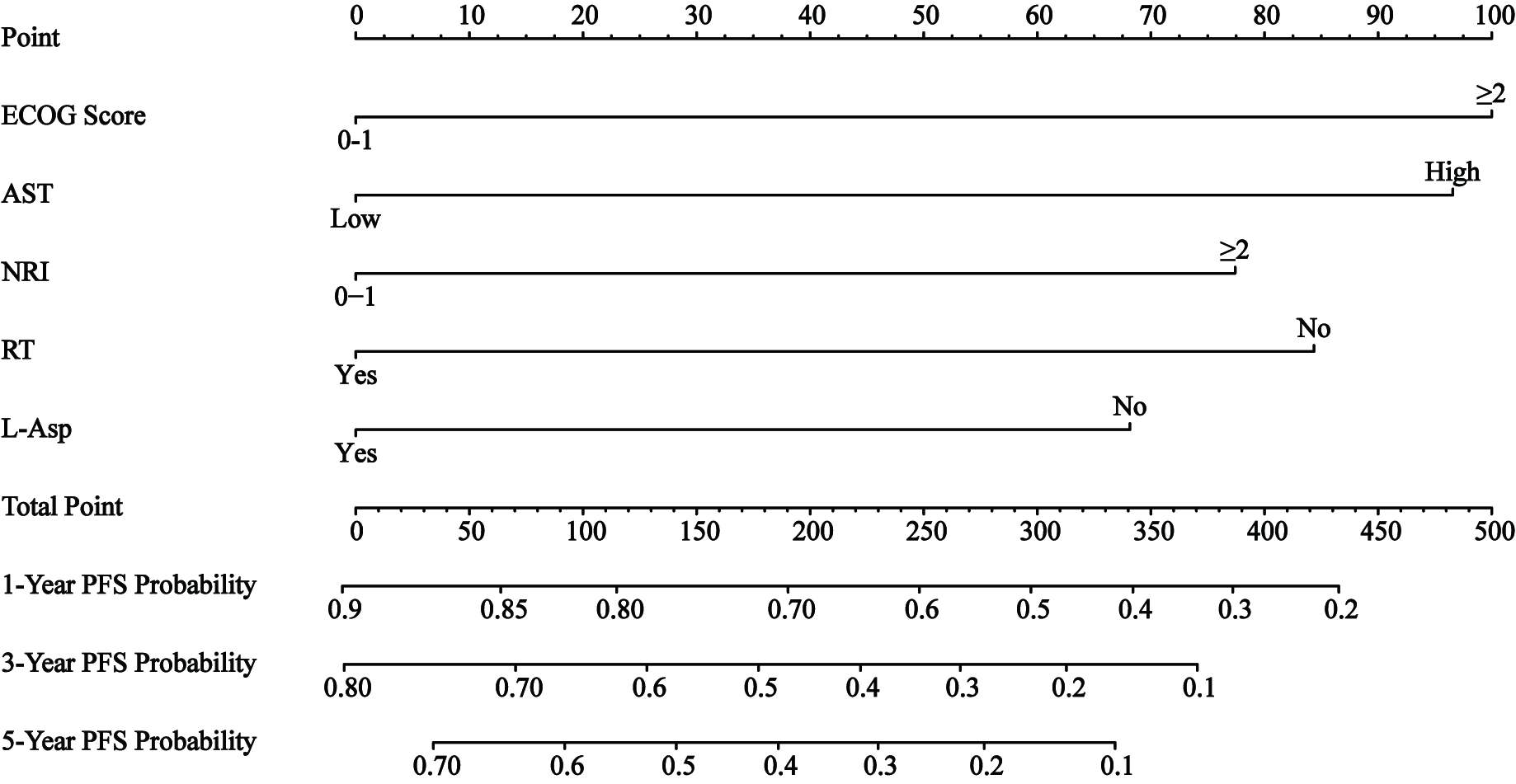

Supplement: Supplementary table and Figure [file cbm-39-cbm230068-s001.docx]
